# Antigenicity of soluble HIV gp140 trimers reveals differences in solution conformation for differing HIV strains

**DOI:** 10.1186/1742-4690-9-S2-P313

**Published:** 2012-09-13

**Authors:** TL Gearhart, JD Steckbeck, RC Montelaro, JK Craigo

**Affiliations:** 1 University of Pittsburgh School of Medicine, Pittsburgh, PA, USA

## Background

Eliciting effective antibody responses are key to the design of HIV vaccines. While the HIV envelope protein is highly immunogenic and provokes a high-titer antibody response during viral infection and experimental immunization, affinity-matured antibodies capable of neutralizing diverse HIV isolates are rarely elicited. While recent vaccine regimens have focused on DNA, viral vector, VLP, or attenuated virus vaccines, of increasing interest are improved recombinant envelope protein immunogens. Immunizations with trimeric gp140 proteins induce higher-titer neutralizing antibody responses and have structural benefits over monomeric gp120 immunizations. Novel approaches being taken for recombinant trimeric gp140 immunogens include the use of consensus and multi-clade gp140 trimers. A consideration for these synthetic proteins that is key to vaccine efficacy: do these proteins structurally represent a native trimeric envelope?

## Methods

To address potential differences in the functional conformation of gp140 trimers, we evaluated the conformational characteristics of trimeric gp140 proteins from varying HIV-1 strains as well as engineered consensus gp140s via binding studies using surface plasmon resonance with a panel of well-characterized monoclonal antibodies (MAbs).

## Results

Consensus trimers were recognized by more monoclonal antibodies than the primary strain gp140s, suggestive of the benefits of engineered trimers. However, analysis revealed a low trimer concentration that is competent to bind the quaternary-recognizing MAb PG9. Considering the binding to other conformational MAbs, this suggests that while the consensus gp140s contain appropriately conformational monomers and are trimeric, the vast majority of the protein is not in a proper quaternary structure.

## Conclusion

These data suggest that it is important to fully assess structural differences of immunogens even though obvious phenotypic differences may not be present. Taken together, these observations demonstrate the need to evaluate immunogens in a manner that allows the measurement of functional epitope exposure and solution conformation to assess the potential to elicit a potent, broadly-neutralizing antibody response.

